# Quality of lower limb preoperative skin preparation using colorless versus colored disinfectants–results of an experimental, randomized study in a close to reality setting

**DOI:** 10.1371/journal.pone.0282662

**Published:** 2023-03-02

**Authors:** Karsten Fink, Marcus Örgel, Claas Baier, Vesta Brauckmann, Vasilis Giannoudis, Emmanouil Liodakis

**Affiliations:** 1 Department of Trauma Surgery, Hannover Medical School, Hannover, Germany; 2 Institute for Medical Microbiology and Hospital Epidemiology, Hannover Medical School, Hannover, Germany; 3 Academic Department of Trauma & Orthopaedics, School of Medicine, University of Leeds, Leeds General Infirmary, Leeds, United Kingdom; Justus Liebig University Giessen, GERMANY

## Abstract

**Background:**

Appropriate preoperative skin cleansing is important to control surgical site infections. Both colored and colorless skin disinfectants are available, however certain types of skin preparation, such as octenidine-dihydrochloride with alcohol have a long residual antimicrobial effect but are available only in colorless form. We hypothesized that colorless skin disinfectants lead to more incomplete skin preparation of lower limbs compared to colored agents.

**Methods:**

We randomly assigned healthy volunteers to undergo a determined skin cleansing protocol for total hip arthroplasty in the supine position to either a colored or colorless skin cleansing protocol. The adequacy of skin preparation was compared between orthopedic consultants and residents. The colorless disinfectant was mixed with a fluorescent dye and missed skin areas were visualized using UV lamps. Both preparations were photo-documented following standardized protocols. The primary outcome of interest was the number of legs with an incomplete scrubbed area. The secondary outcome was the cumulative skin area not disinfected.

**Results:**

Fifty-two healthy volunteers (104 legs; 52 colored and 52 colorless) underwent surgical skin preparation. The number of legs incompletely disinfected was significantly higher in colorless compared to colored disinfectant group (38.5% (n = 20) vs. 13.5% (n = 7); p = 0.007). Regardless of the disinfectant, consultants performed better than the residents. When using colored disinfectant, residents incompletely prepared the site in 23.1% (n = 6) compared with 57.7% (n = 15) with a colorless disinfectant (p = 0.023). Conversely consultants using colored disinfectant incompletely prepared the site in 3.8% (n = 1) compared with 19.2% (n = 5) for colorless disinfectant (p = 0.191). The total amount of uncleansed skin was significantly higher using colorless skin disinfectant (mean ± standard deviation: 8.78 cm^2^± 35.07 vs. 0.65 cm^2^ ± 2.66, p = 0.002).

**Conclusions:**

Application of colorless skin disinfectants for hip arthroplasty cleansing protocol led to decreased skin coverage among consultants and residents compared to colored preparations. Colored disinfectants remain the gold standard in hip surgery, however we should be aiming to develop newer colored disinfectants with long residual antimicrobial effects to enable visual control during the scrubbing process.

## Introduction

Surgical site infections (SSI) can occur from 30 days postoperatively to within 1 year post surgery, if implants are used [1]. Preoperative use of antimicrobial prophylactic agents is part of the prevention of an early SSI [2]. In orthopedic surgery, SSIs are most commonly associated with implants (peri-implant infection or periprosthetic infection in arthroplasties; (PPI)). Due to the formation of biofilms accompanied by acute and chronic infection processes, an aggressive surgical debridement, partial or complete change of the implant material and a targeted antibiotic therapy are necessary [3]. Despite a clinical curative rate ranging from 79% - 100% [4], PPIs have fulminant individual and socio-economic consequences [5,6]. Depending on the respective health care system and the type of SSI, the costs can vary. For instance, in the US, the costs can exceed $ 96,166 ± 60,664 per infection when the SSI involves a prosthetic joint implant [7,8]. SSIs are among the more common complications of joint replacement surgery, arising in 0.2% to 2% of patients, or as many as 9% in special situations such as the implantation of so-called megaprostheses [9]. The preoperative skin preparation of the surgical field using skin disinfectants is a major cornerstone in the prevention of surgical site infections (SSIs) [10,11]. Alcoholic disinfectants are the first choice for preoperative skin preparation in orthopedic surgery because of their rapid and broad-spectrum antibacterial effect. However, sole alcohol (e.g., Isopropylalcohol, IPA) based skin preparations do not have a residual antibacterial effect [12,13]. Therefore, additives such as chlorhexidine (CHX), povidone-iodine (PVP-I) or octenidine dihydrochloride (OCT) are used to gain such a residual long-term antibacterial effect. Noteworthy, the superiority of PVP-I + alcohol or CHX + alcohol over PVP–I alone (in aqueous solution), in terms of effectively reducing SSIs, has been shown—in several randomized multicenter trials, across various medical disciplines [14–18]. Tuuli and colleagues conducted a trial comparing CHX + IPA and PVP-I + IPA in comparable alcoholic formulations for skin disinfection with superiority for CHX + IPA [19]. Based on the current data, CHX and OCT appear to be very good agents in terms of residual effects [20–22]. The German company Schülke (Schülke & Mayr, Norderstedt, Germany), the manufacturer of Octeniderm® (a alcohol-based colorless disinfectant with OCT which is often used in Germany) describes a residual effect of 48 hours. However, this product cannot be dyed without lowering its antimicrobial effect. As the quality of the preoperative skin preparation is also influenced by the completeness of the application on the skin within the surgical field and the modes of application [23] colored disinfectants are often preferred in orthopedic surgical practice. They enable quick, accurate and easy visual feedback.

### Objectives

We conducted an experimental study comparing the skin coverage with a colored and a colorless preparation following a standardized protocol in simulated total hip arthroplasty (THA) cases. Skin disinfection for THA in the supine position can be challenging due to the large and complex surgical field with some skin areas often being out of direct sight. Furthermore, we investigated the effect of the surgeons’ clinical experience level on the disinfection quality. Our hypothesis was that the use of colorless disinfectants leads to incomplete skin coverage of the scrubbing area and that the surgeon’s experience may also play a role.

## Materials and methods

We conducted this randomized controlled clinical, single-centre trial between March and September 2021 at the Hannover Medical School (MHH). No changes to the methods were made after trial start. The local ethical committee approved the study (Nr.: 9579_BO_S_2021), and written informed consent was obtained from all healthy volunteers participating in the study before recruitment. The manuscript follows the CONSORT guidelines for reporting parallel group randomized trials and corresponds to the CONSORT checklist [24,25].

### Participants

Volunteers were recruited during clinical contact and in the context of student teaching. The key inclusion criteria were healthy adults aged between 18 and 65 years old with a body mass index of under 30 kg/m^2^. The skin color should be typically fair, medium-fair or medium-dark according to the white average of the central European population. Exclusion criteria were a history of allergy to alcohol, OCT, CHX or PVP-I, any excipients, or participants with scars, large burns or lesions on the legs that may impact the study. We chose 52 healthy volunteers to participate in this study, 26 women and 26 men. The average age was 23.37 ± 3.54 years (min. 19, max. 27) with a mean BMI of 22.1 ± 1.3 kg/m^2^ (min 18.7, max. 23.9). Scrubbing always occurred in a room without windows and a common stretcher was used. In cooperation with the biometrics department, the study was first conducted on 10 subjects and then a power analysis was carried out to determine the number of cases required which led to the participant count of 52.

### Randomization and cleansing protocol

**Fig 1:**
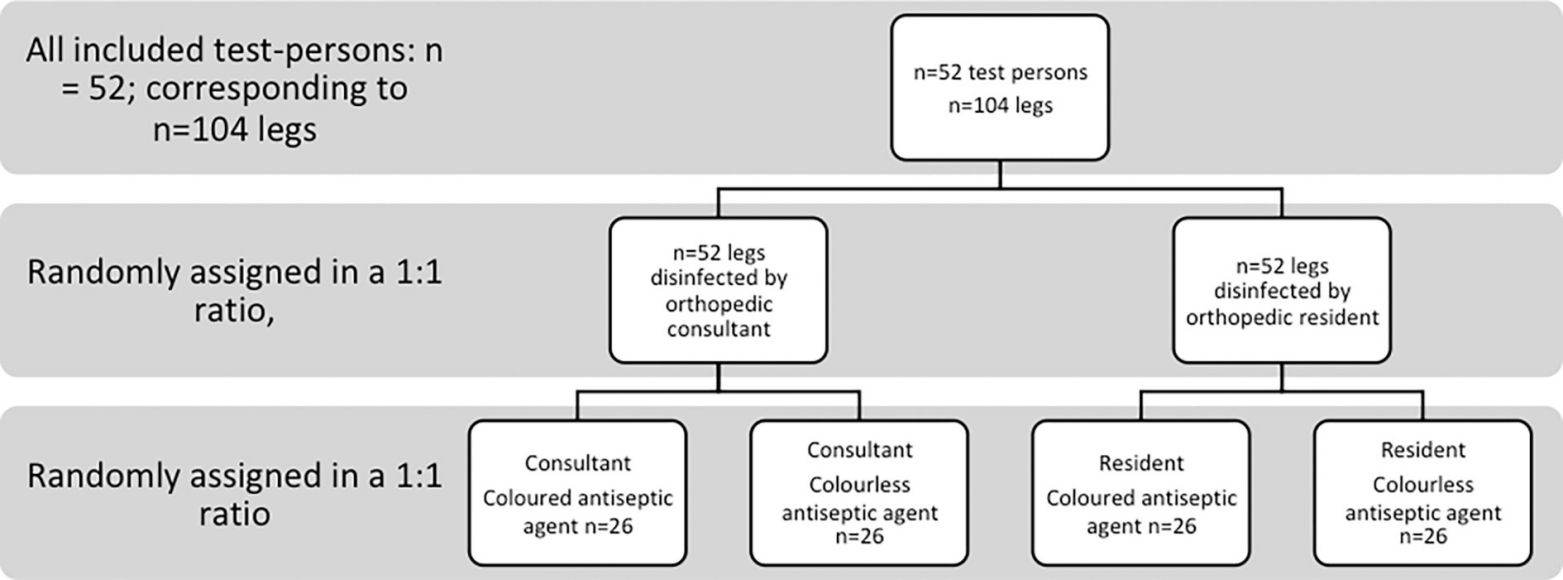
Randomization and treatment regime used in our study.

An orthopaedic resident or an orthopaedic consultant were randomly assigned in a 1:1 ratio. They were randomly asked to scrub the right or left leg of the volunteer and to start with a colored or colorless antiseptic, as shown in the chart (Fig 1). Randomization was performed using web-based randomization service (https://www.randomizer.org). This randomization was used by an independent investigator to allocate the cleansing protocol. The color of the solution and therefore the participants could not be fully blinded, however the aim of the study was not communicated.

**Fig 2:**
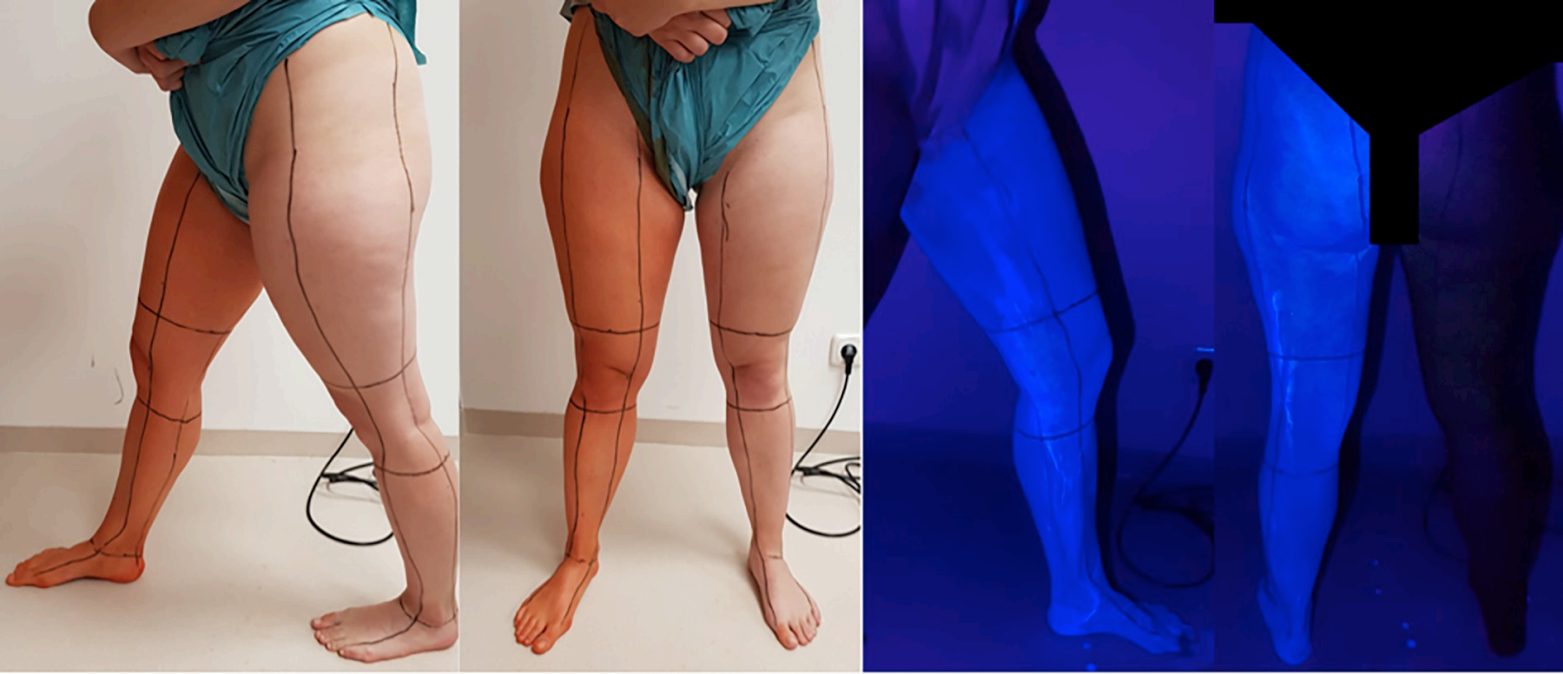
The leg was divided into 17 zones as demonstrated on the two images to the left: 1) thigh ventral, 2) thigh lateral, 3) thigh dorsal, 4) thigh medial, 5) knee ventral, 6) knee lateral, 7) knee dorsal, 8) knee medial, 9) lower leg ventral, 10) lower leg lateral, 11) lower leg dorsal, 12) lower leg medial, 13) foot ventral, 14) foot lateral, 15) foot plantar, 16) foot medial, 17) heel. The two images on the right show the appearance of the skin preparation under the UV light.

A commercially available alcohol + PVP-I–based skin disinfectant (Braunoderm®, B. Braun, Melsungen, Germany) was used as a colored disinfectant, and Schülke-Optics (Schülke & Mayr, Norderstedt, Germany) was used as a colorless solution. Schülke-Optics contains among other ingredients IPA and is used for training and for review because it can be visualized using an UV lamp under darkened conditions (Fig 2). The cleansing protocol was standardized using a swab tong and 12 swabs for each leg. In-house standard with back-and-forth movements was the application method of choice. The leg was divided into 4 zones: the upper thigh, knee, lower leg and foot with the heel. For each zone, 3 swabs were used.

The cleansing procedure started ventral. The sequence was: 1) the ventral thigh, 2) the lateral thigh, 3) the dorsal thigh, and 4) the medial thigh. The same sequence was performed for the zones around the knee, lower leg and foot. For the foot, the heel was considered to be an additional zone. The leg was held at the heel/ lower leg by a second person and the investigator switched sides during cleansing protocol. The surgeons were directly informed before the intervention, which disinfectant and which side they had to start with. No sterile drapes were used.

### Outcomes

The primary outcome of interest was the number of legs with an incomplete scrubbed area.

An incomplete skin cleansing area was defined as any visibly not disinfected skin part regardless of size. The secondary outcome was the cumulative size of these areas measured in square centimeters.

### Assessment

Following skin disinfection, a fixed sequence of photographs was performed for documentation purposes. These photographs were systematically reviewed by an independent investigator and missed skin areas were documented, lined out and the area was measured in square centimeters.

**Fig 3:**
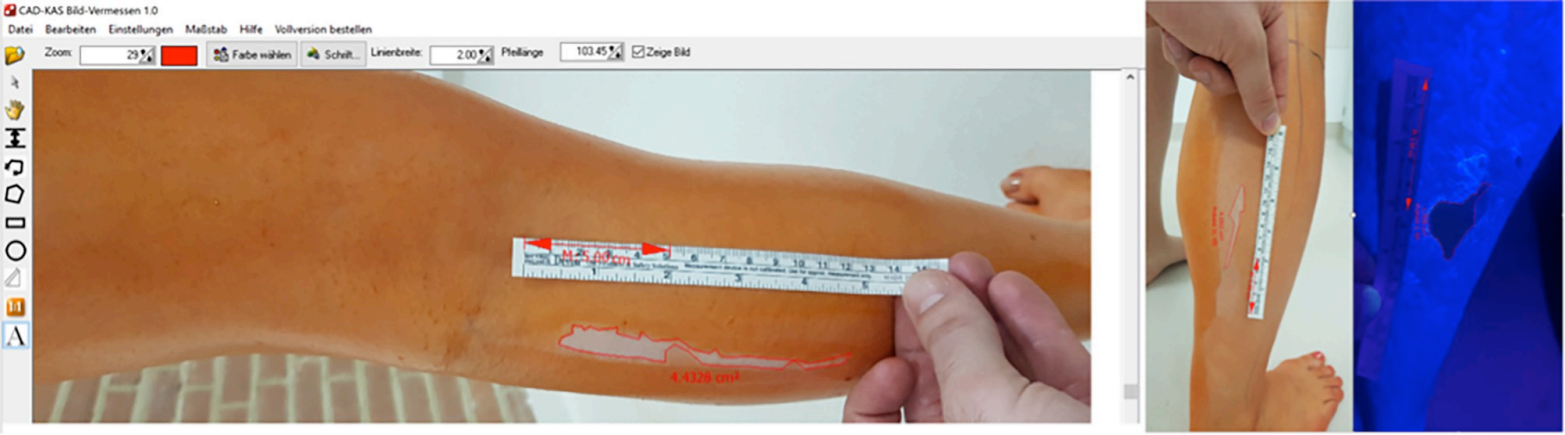
Figure demonstrating the areas missed and marked out through the use of the CAD-KAS system.

For this we used the “CAD-KAS Bild-Vermessen 1.0 software (CAD-KAS Kassler Computersoftware GbR, Markranstädt, Germany, UID: DE202044533) (Fig 3). In order to assign the forgotten skin areas to the exact anatomical site, the leg was divided into 17 areas (Fig 2). Thigh ventral/lateral / dorsal / medial, knee ventral/lateral / dorsal / medial, lower leg ventral / lateral / dorsal / medial, foot ventral / lateral / plantar / medial and heel, like in the figure above.

### Statistical analysis

Continuous variables were checked for normal distribution using the Shapiro–Wilk test, and are presented as mean ± standard deviation (SD). Categorical variables were described as frequencies with percentages. The primary data analyses followed the intention-to-treat principle, in which data from all participants were analysed in the group to which the participants were randomly assigned, regardless received intervention [24]. The significance of differences between the study groups was determined with the Mann–Whitney U test for nonparametric continuous variables. Since the one leg was disinfected with colorless agents and the contralateral with colored agents, the samples (colored vs. colorless) were deemed to be independent since a differing leg was prepared by the investigator, so an unpaired t-test was used. For categorical variables the Chi square and Fisher’s exact test were used according to the sample size. A two-tailed p-value of ≤ 0.05 was considered statistically significant. The SPSS 27.0 program (SPSS Inc., Chicago, IL) was used for statistical analyses.

## Results

**Fig 4:**
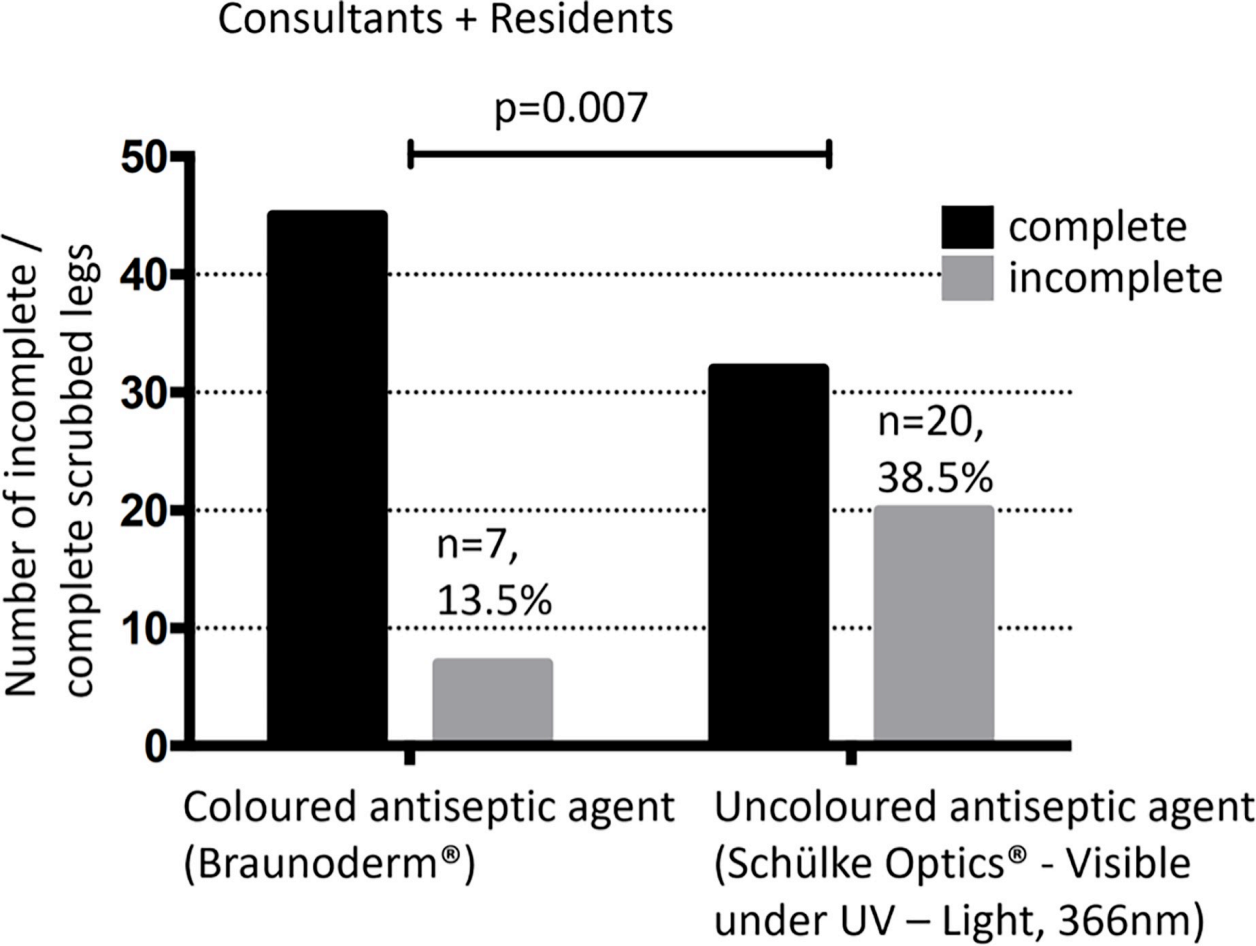
Graph combining the resident and consultant skin preparation completeness based on the number of legs found to have been disinfected fully.

The members of the two study groups were all medical students. They were similar with respect to demographic characteristics such as: age, weight and height. The ratio of women to men was 1:1. The overall percentage of uncleansed skin areas was significantly higher in the colorless disinfectant group compared with the colored disinfectant group (38.5% vs. 13.5%; p = 0.007) (Fig 4).

**Fig 5:**
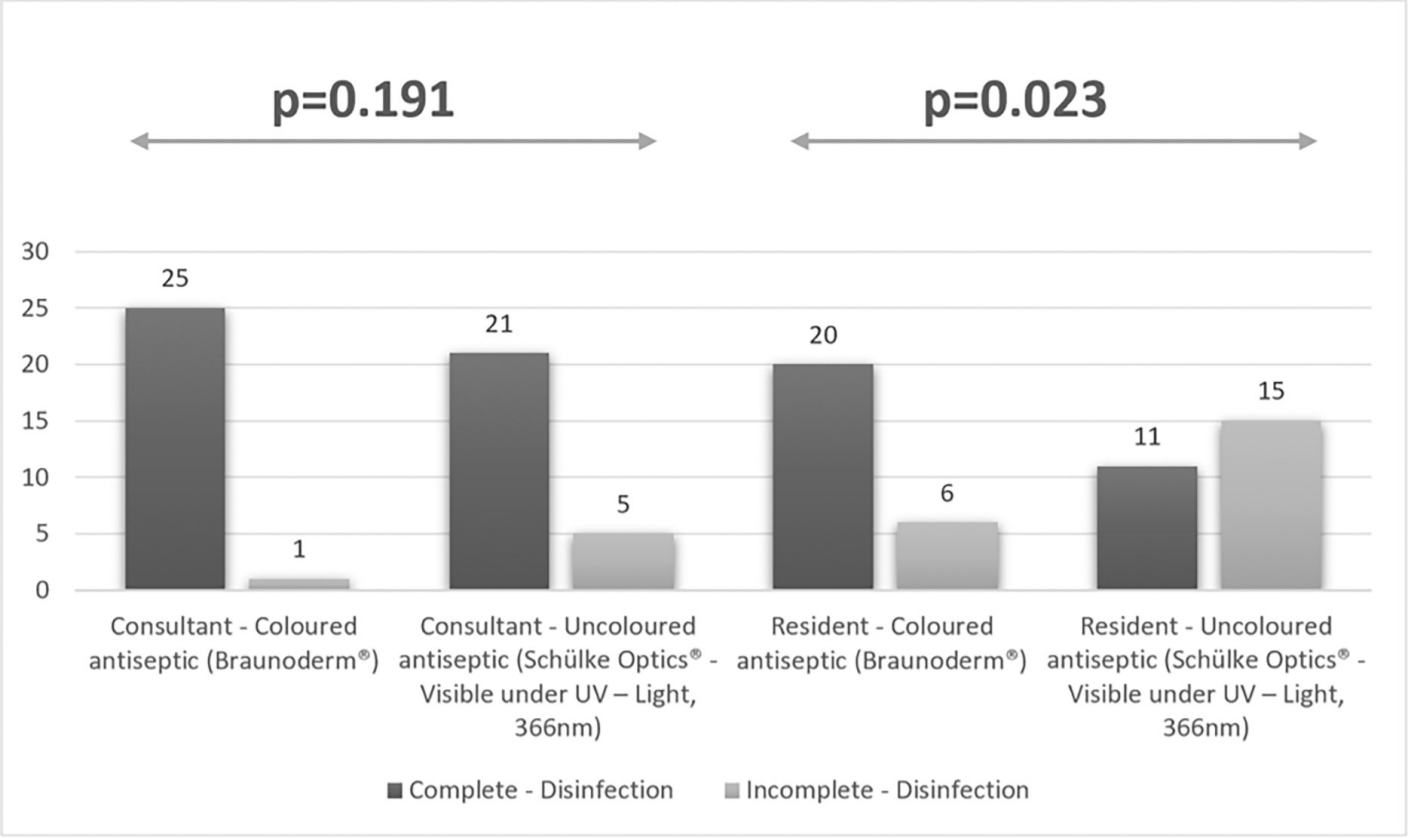
Comparison between consultant and resident THA skin preparation when using colored vs. colorless skin antiseptic.

When comparing the use of colored and colorless disinfectants between consultants there was no statistically significant difference. In 26 legs disinfected by consultants using colored antiseptics, only one leg was partially disinfected (3.8%). In 26 legs disinfected by consultants using colorless antiseptics, five legs were disinfected partially (19.2%) p-value = 0.191 (*Fig 5*).

When comparing the use of colored and colorless disinfectants between residents there was a statistically significant difference. In 26 legs disinfected by residents using colored antiseptics, six legs were disinfected partially (23.1%). In 26 legs disinfected by residents using colorless antiseptics, 15 legs were disinfected partially (57.7%) p-value = 0.023 (*Fig 5*).

**Fig 6:**
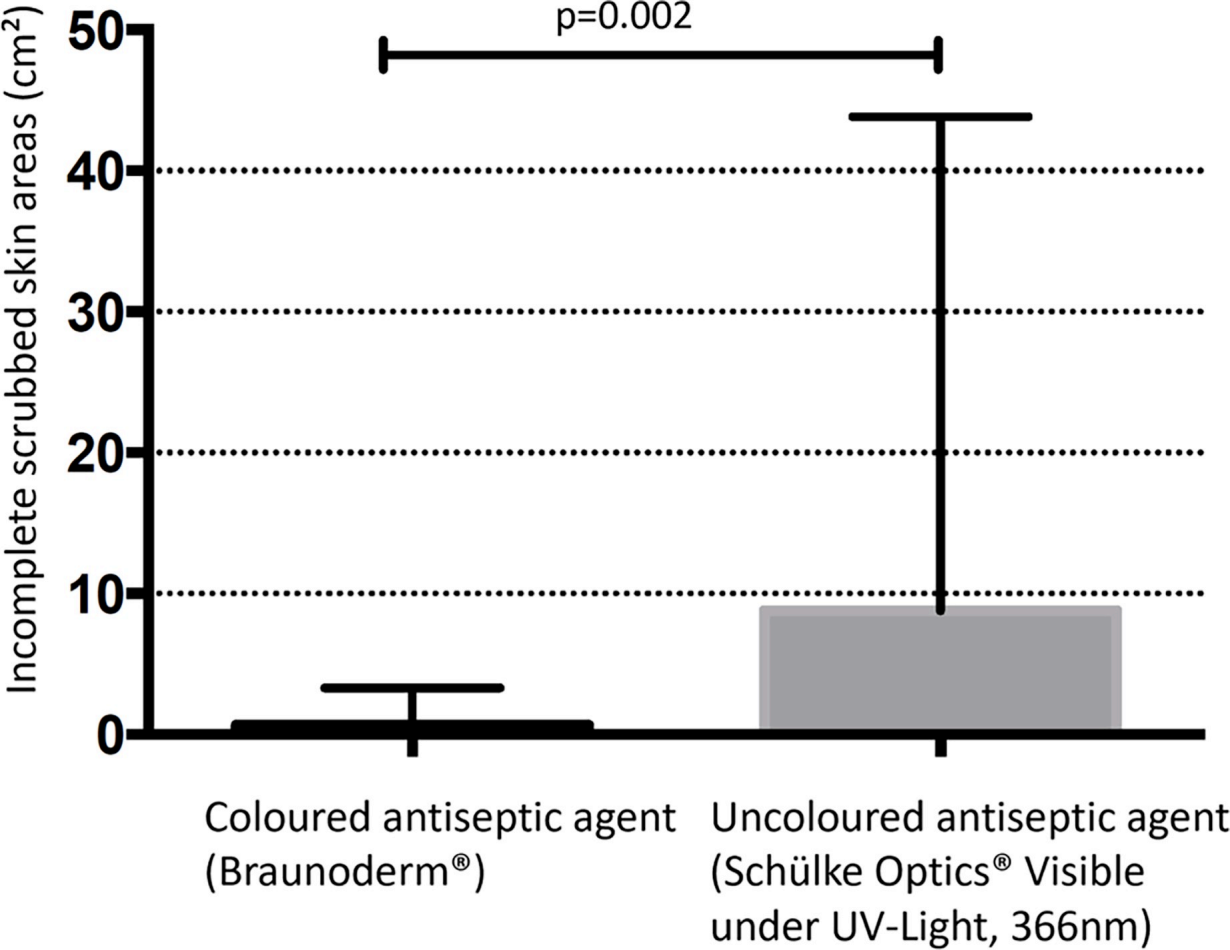
Graph demonstrating the difference in surface area of inadequately prepared skin (Colored antiseptic v.s. colorless antiseptic).

We assumed the human body surface as being 1.8m^2^ by using the Dubois formula [26] and our average BMI. We then calculated the percentage surface area of a human leg (18% of body surface area) according to "Wallace rule of nine” [27] equating to 0.324m^2^ per leg. The sum of the non-disinfected areas when using colorless antiseptics was 456.56 cm^2^, whilst when using colored antiseptic was 33.8 cm^2^. The total amount of uncleansed skin in square centimeters was significantly higher using colorless skin disinfectant (mean ± standard deviation: 8.78 cm^2^± 35.07 vs. 0.65 cm^2^ ± 2.66, p = 0.002) (*Fig 6*). According to our data, the surface in square centimeters of uncleansed skin was higher by the factor of 13.5 when using colorless skin antiseptic. This equates to 1.04% of a leg being inadequately scrubbed by using a colored antiseptic compared to 14.1% when using uncolored antiseptic (Fig 6).

Dividing this data by the level of seniority of surgeon (consultant vs. resident) showed that the area of uncleansed skin was significantly smaller when prepped by a consultant.

The total amount of uncleansed skin in square centimeters by residents using colorless vs. colored skin disinfectant was 16.10±47.89 vs. 1.29±3.68, (p = 0.006). In contrast, the total amount of uncleansed skin in square centimeters by consultants using colorless vs. colored skin disinfectant was 0.84±1.92 vs. 0.01±0.07 (p = 0.072).

The forgotten skin areas were more often located outside the field of view of the surgeon. This data was not statistically significant; however, we can infer from this that attention must be paid to the dorsal portion of the leg (Table 1).

**Table 1:** Outcome data.

	Colored legs overall (n = 52)	Colorless legs overall (n = 52)	p-value	Colored legs by resident (n = 26)	Colorless legs by resident (n = 26)	p-value	Colored legs by consultant (n = 26)	Colorless legs by consultant (n = 26)	p-value
**Thigh**									
**• ventral**	0.00±0.00	0.00±0.00	-	0.00±0.00	0.00±0.00	-	0.00±0.00	0.00±0.00	-
**• lateral**	0.00±0.00	0.00±0.00	-	0.00±0.00	0.00±0.00	-	0.00±0.00	0.00±0.00	-
**• medial**	00.00±0.00	1.49±5.94	0.006	00.00±0.00	2.53±8.18	0.020	00.00±0.00	0.45±1.67	0.153
**• dorsal**	00.00±0.00	0.01±0.07	0.317	0.00±0.00	0.02±0.10	0.317	0.00±0.00	0.00±0.00	-
**Knee**									
**• ventral**	0.00±0.00	0.00±0.00	-	0.00±0.00	0.00±0.00	-	0.00±0.00	0.00±0.00	-
• **lateral**	0.00±0.00	0.00±0.00	-	0.00±0.00	0.00±0.00	-	0.00±0.00	0.00±0.00	-
• **medial**	0.00±0.00	0.12±0.82	0.155	0.00±0.00	0.24±1.13	0.153	0.00±0.00	0.00±0.00	-
• **dorsal**	0.00±0.00	1.98±11.74	0.08	0.00±0.00	3.96±16.52	0.077	0.00±0.00	0.00±0.00	-
**Lower leg**									
• **ventral**	0.00±0.00	0.00±0.00	-	0.00±0.00	0.00±0.00	-	0.00±0.00	0.00±0.00	-
• **lateral**	0.00±0.00	0.00±0.00	-	0.00±0.00	0.00±0.00	-	0.00±0.00	0.00±0.00	-
• **medial**	0.00±0.00	1.21±6.13	0.155	0.00±0.00	2.43±8.58	0.153	0.00±0.00	0.00±0.00	-
• **dorsal**	0.59±2.32	3.47±17.85	0.300	1.17±3.21	6.73±25.05	0.560	0.01±0.07	0.21±0.65	0.276
**Foot**									
• **ventral**	0.00±0.00	0.23±1.15	0.080	0.00±0.00	0.45±1.61	0.077	0.00±0.00	0.00±0.00	-
• **lateral**	0.01±0.08	0.01±0.09	0.989	0.02±0.12	0.03±0.13	0.978	0.00±0.00	0.00±0.00	-
• **medial**	0.03±0.22	0.24±0.77	0.050	0.06±0.31	0.32±0.92	0.163	0.00±0.00	0.18±0.61	0.153
• **plantar**	0.01±0.07	0.00±0.00	0.317	0.02±0.10	0.00±0.00	0.317	0.00±0.00	0.00±0.00	-
• **heel**	0.01±0.04	0.00±0.00	0.317	0.01±0.06	0.00±0.00	0.317	0.00±0.00	0.00±0.00	-

## Discussion

We hypothesized that the visualization of skin antiseptics is an important factor in orthopaedic joint surgery. Surgical skin preparation of a complete leg in THA is more challenging than for example abdominal/spinal surgery because the areas are much larger, three-dimensional and sometimes outside one’s own field of view, we were able to demonstrate the following in our study:

1. Using colored antiseptic disinfection in THA (supine) leads more often to complete skin coverage, than colorless, as demonstrated by overall adequacy of skin preparation (overall view: 13.5% vs. 38.5%. level of significance: p = 0.007).
2. The combination of resident orthopedic surgeons and use of colorless antiseptic agent provided the highest rate of incomplete skin preparation. It is often common practice that the resident starts with the preoperative skin preparation, before the consultant joins for the surgical part.
3. There is no control function by the operating theatre staff when using uncolored disinfectants.
4. Even when incomplete skin areas appeared after skin preparation, the size was significantly smaller when using the colored antiseptic agent.
5. Most of the time uncleansed skin areas were outside the surgeon view, like dorsal lower leg. So at least one additional person in the operating room should always observe the disinfection process.

One limitation of this study was our inability to blind the surgeons or the examiners of the photo documentation regarding which antiseptic agent used, as colored vs. colorless skin preparation was easy to distinguish between. Furthermore, another limitation of our study was the lack of heterogeneity in our study group. Most participants were young and healthy and as a consequence the composition of their body and skin (e.g., skin laxity) may well be different in our elderly population. We attempted to minimized the impact of this limitation through not telling study participants what the aims of the study were.

The study strength is that for the first time, the actual skin coverage of colorless antiseptic agent, in supine THA protocol could be visualized, by using UV—light.

Several studies have investigated different skin disinfectants with respect to the risk of subsequent SSI. PVP-iodine (in aqueous solution) alone is clearly inferior to reduce surgical site infections, which has been shown in various studies and meta-analysis (14, 18). Although there is some evidence that CHX + alcohol is superior to PVP-I + alcohol, proof in orthopedic hip surgery is still pending [17,19]. The WHO recommends in its global guideline the use of alcohol-based disinfectants with CHX [11]. From a microbiological point of view, regarding the residual antibacterial effect, besides CHX also OCT can be considered to be a good disinfectant [28], for instance in combination with alcohol [29,30]. However, at the moment, no available commercial product with alcohol + OCT is tinted (product Octeniderm® of the company Schülke) [20–22,29,30]. Colored CHX + alcohol based skin disinfectants are available on the market and well-established (ChloraPrep™). Furthermore, there is evidence underlining a correlation between well-rehearsed surgical teams and the occurrence of SSI [31–34]. This is to some extent reflected in our finding that consultants perform better than residents.

## Conclusions

We conclude that colored disinfectants remain the gold standard for skin preparation in hip surgery. The aim should be to use available, clearly visible, colored, alcohol-based disinfectants that also have residual antibacterial effect (e.g., due to addition of CHX [28]). In order to comprehensively evaluate the overall performance of a OCT + alcohol based disinfectant for preoperative skin preparation, a tinted preparation should be developed (without lowering its antimicrobial effect) since efficacy depends not only on the composition but also on the adequate application, which is clearly facilitated by the presence of a tint.

## Supporting information

S1 Checklist CONSORT 2010 checklist of information to include when reporting a randomised trial*. (PDF)

S1 Dataset (XLSX)
